# Ultrasound-guided pleural puncture in supine or recumbent lateral position - feasibility study

**DOI:** 10.1186/2049-6958-8-18

**Published:** 2013-03-13

**Authors:** Gino Soldati, Andrea Smargiassi, Riccardo Inchingolo, Sara Sher, Salvatore Valente, Giuseppe Maria Corbo

**Affiliations:** 1 Emergency Medicine Unit, Castelnuovo Garfagnana General Hospital, Lucca, Italy; 2 Pulmonary Medicine Department, University Hospital A. Gemelli, Rome, Italy; 3 Anesthesia and Intensive Care Unit, Niguarda Cà Granda Hospital, Milan, Italy

**Keywords:** Chest drainage, Chest ultrasonography, Pleural effusion, Thoracentesis, Veres needle

## Abstract

**Background:**

The aim of this study is to evaluate feasibility, safety and efficacy of accessing the pleural space with the patient supine or in lateral recumbent position, under constant ultrasonic guidance along the costophrenic sinus.

**Methods:**

All patients with pleural effusion, referred to thoracentesis or pleural drainage from February 2010 to January 2011 in two institutions, were drained either supine or in lateral recumbent position through an echomonitored cannulation of the costophrenic sinus. The technique is described in detail and an analysis of safety and feasibility is carried out.

**Results:**

One hundred and one thoracenteses were performed on 76 patients and 30 pigtail catheters were inserted in 30 patients (for a total of 131 pleural procedures in 106 patients enrolled). The feasibility of the procedures was 100% and in every case it was possible to follow real time needle tip passage in the pleural space.

Ninety eight thoracenteses (97%) and all catheter drainages were successfully completed. Four thoracenteses were stopped because of the appearance of complications while no pigtail drainage procedure was stopped. After 24 hour follow up, one chest pain syndrome (1.3% of completed thoracenteses) and two pneumothoraces (1.4%) occurred. The mean acquisition time of pleural space was 76 ± 9 seconds for thoracentesis and 185 ± 46 seconds for drainage insertion (p < 0.05).

**Conclusions:**

This study highlights the safety and efficacy of this technique of real time echo-monitored pleural space puncture, that offers a more comfortable patient position, an easier approach for the operator, a very low rate of complications with short acquisition time of pleural space.

## Background

Over the last several years, the use of portable ultrasound machines has greatly enhanced the evaluation and the operative management of many pleural diseases [1]. The advantages of ultrasound guided procedures on the chest include: the absence of ionizing energies, the sensitivity of ultrasound for the detection of pleural fluid and the possibility of acquiring real time images.

Pleural effusion is a commonly encountered clinical entity and most patients are going to undergo thoracentesis or tube thoracostomy to define the aetiology of the pleural effusion and/or to relieve symptoms. These invasive procedures can be performed with or without the aid of diagnostic imaging.

Generally, ultrasound guidance is indicated if some difficulties are predictable, if a problem is encountered during the procedure or if the pleural effusion is small and/or loculate, and the use of ultrasound is often limited to marking the best insertion point, to mechanically ventilated patients and after a failed clinically directed thoracentesis [2-5]. However recently the ultrasound guided pleural puncture is gaining the role of a standard procedure [6].

In these cases, as in the classical blind access technique, pleural puncture is carried out with direct parietal crossing and with the needle pointing in a 90° direction with respect to lung parenchyma.

Although there are no blinded randomized studies comparing physical examination guided (blind) thoracentesis and ultrasound guided thoracentesis, several trials have shown lower rate of complications with sonographic guidance [3,7]. Consequently, the interest of many physicians (pulmonary medicine, critical care, surgery, emergency medicine) for chest interventional sonography is growing.

Another field of study is the development of dedicated procedural techniques for a safe thoracentesis, properly designed for an ultrasonographic approach. Most studies on this topic, in fact, do not use real time guidance for needle insertion, but they use ultrasound scanning for the identification of an appropriate puncture site (“X marks the spot” or ultrasound ‘oriented’ technique) [2-4,7] without considering the correct scanning plane relative to puncture nor an elective puncture site, thus missing the benefits of real time visualization of needle progression in the pleural cavity.

In our opinion, the choice of an elective puncture site in terms of operative approach and patient positioning, the possibility of directly observing needle progression, echoguided fluid aspiration and controlled needle withdrawal, could avoid accidental injuries of parietal and parenchymal tissues and allow more complete fluid drainage.

The aim of this study was to develop a needle insertion technique under total ultrasonographic guidance along the costophrenic sinus. In order to evaluate procedural safety and efficacy, we enrolled a series of patients referred to ultrasound guided pleural drainage and both feasibility and complications were analyzed.

## Methods

From February 2010 to January 2011 all patients referred to thoracentesis in the Emergency Department, Valle del Serchio General Hospital, Lucca, and Pulmonary Medicine Department, “A. Gemelli” University Hospital, Rome, Italy, were enrolled in the study. The study received the approval of the Internal Review Board (Ethical Committee of Catholic University of Sacred Heart, Rome, approval number: A.1278/C.E./2010) and every patient gave written informed consent.

All patients received echographic lung scanning for diagnosis, on the basis of clinical grounds and for effusion characteristics and quantification estimate. The indication to thoracentesis was diagnostic, therapeutic or both, while a pigtail catheter was placed in presence of corpusculated, septic, neoplastic or hematic pleural fluid and in any case of large (estimated to be over 1,500 ml) or recurrent effusion.

Relevant labs were carried out (INR ratio, platelet count) and eventually corrected (platelets > 50000/μl, INR < 1.5).

A peripheral vein was cannulated for slow saline infusion and heart rate, blood pressure and pulsoximetry were monitored.

Procedures were performed by one of three physicians (two pulmonologists and one emergency medicine specialist) with at least one year experience in clinical ultrasound and at least two months experience in chest interventional sonography.

For all procedures a Toshiba Aplio XV (Toshiba Medical Systems S.r.l., Rome, Italy) or MyLab™ 50 Gold Cardiovascular (EsaoteS.p.a., Rome, Italy) machines with 3.5-5 and 7.5-10 MHz probes were used.

**Figure 1:**
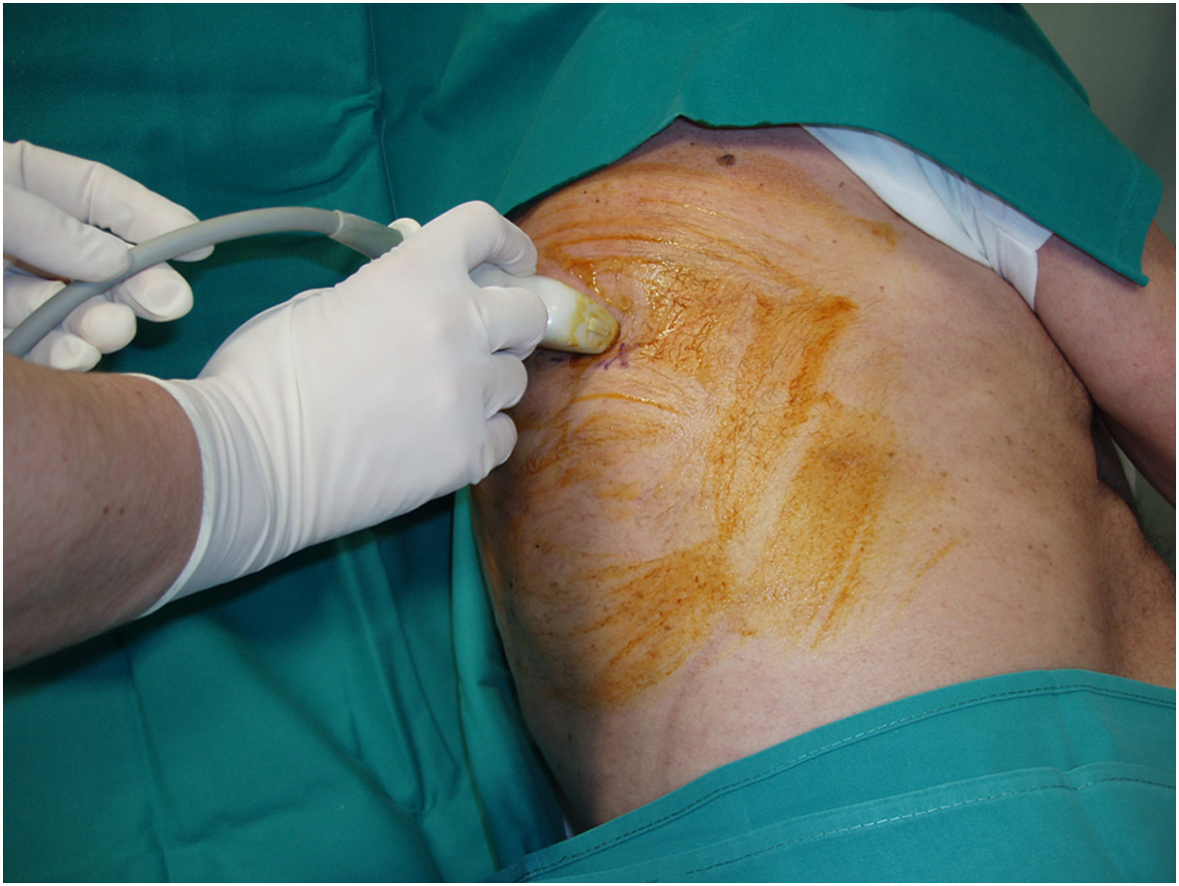
**The patient lies in lateral recumbent position with the side to be drained on top. **The operator localizes the costophrenic sinus in the basal, postero-lateral region of the chest with a Convex probe.

A 3.5-5 MHz convex probe was used to localize fluid collection with the patient sitting or lying supine and the involved side on top. Multiple scans were obtained in both sagittal and intercostal planes. Positive identification of pleural fluid required demonstration of a retroparietal and supradiaphragmatic fluid space with an atelectatic or compressed lung, according to current literature [8]. The costophrenic sinus was identified in its lateral or postero-lateral position (Figure 1).

Pleural effusion was semi-quantitatively evaluated in four grades:

a) Minimal: a pleural effusion limited to the costophrenic sinus scanning plane;

b) Small: an effusion beyond the costophrenic sinus but within one longitudinal scanning plane when using a convex probe set to a depth of 8 cm;

c) Medium: effusion necessitating two contiguous longitudinal scans;

d) Large or Massive: effusion beyond two longitudinal scans at a depth set at 8 cm.

At the preliminary scan effusions were classified as:

a) Transonic: no internal echoes;

b) Complex: corpusculated with echoes in suspension;

c) Fibrinous: when linear branches were present within the fluid;

d) Areolar: spider web aspect of the fluid.

Echographic description was compared with exudative o transudative nature of the fluid as successively determined using Light criteria [9].

There was no formal lower limit of effusion size beneath which thoracentesis was not attempted, but generally a visceral to parietal distance of at least 10 mm in the costophrenic sinus was required.

**Figure 2:**
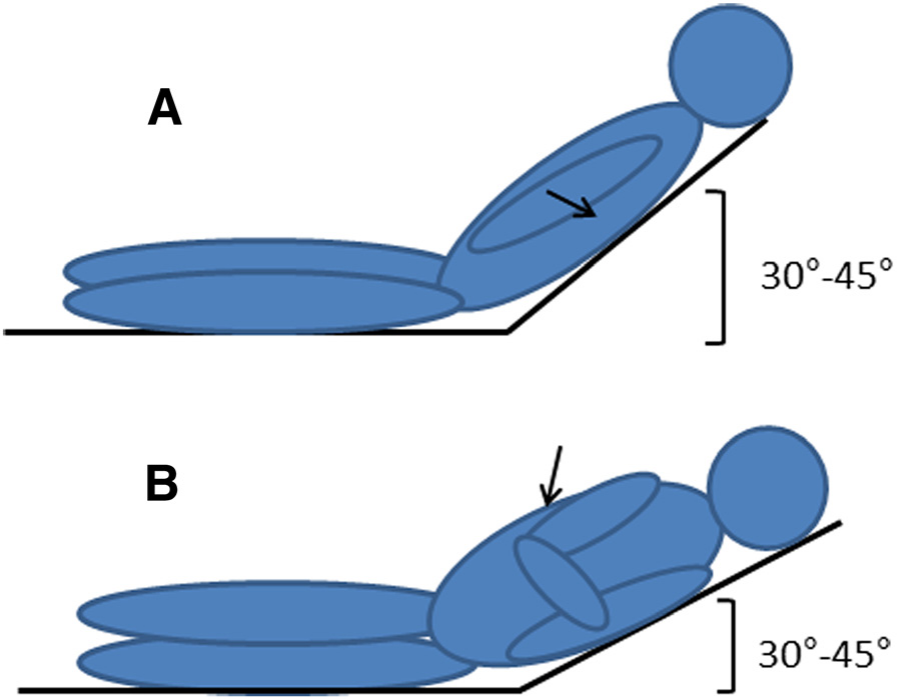
**Patients’ positions described in the text: A**) supine with head and chest elevated at 30-45°; **B**) lateral recumbent with head and chest elevated at 30-45°. Black arrows indicate the site of pleural procedures.

Patients were placed in either supine or opposite lateral recumbent position with head and chest elevated at 30-45° (Figure 2). Pleural fluid over the diaphragm was identified. The cutaneous area was cleaned with povidone iodine. Cutaneous, subcutaneous, muscular, periosteal and parietal pleural anesthesia was carried out under constant ultrasound vision with 1% lidocaine without epinephrine using a 25-gauge spinal needle. A 7.5-10 MHz linear probe was used for anesthesia and for all the real time ultrasound guided interventional procedures.

**Figure 3:**
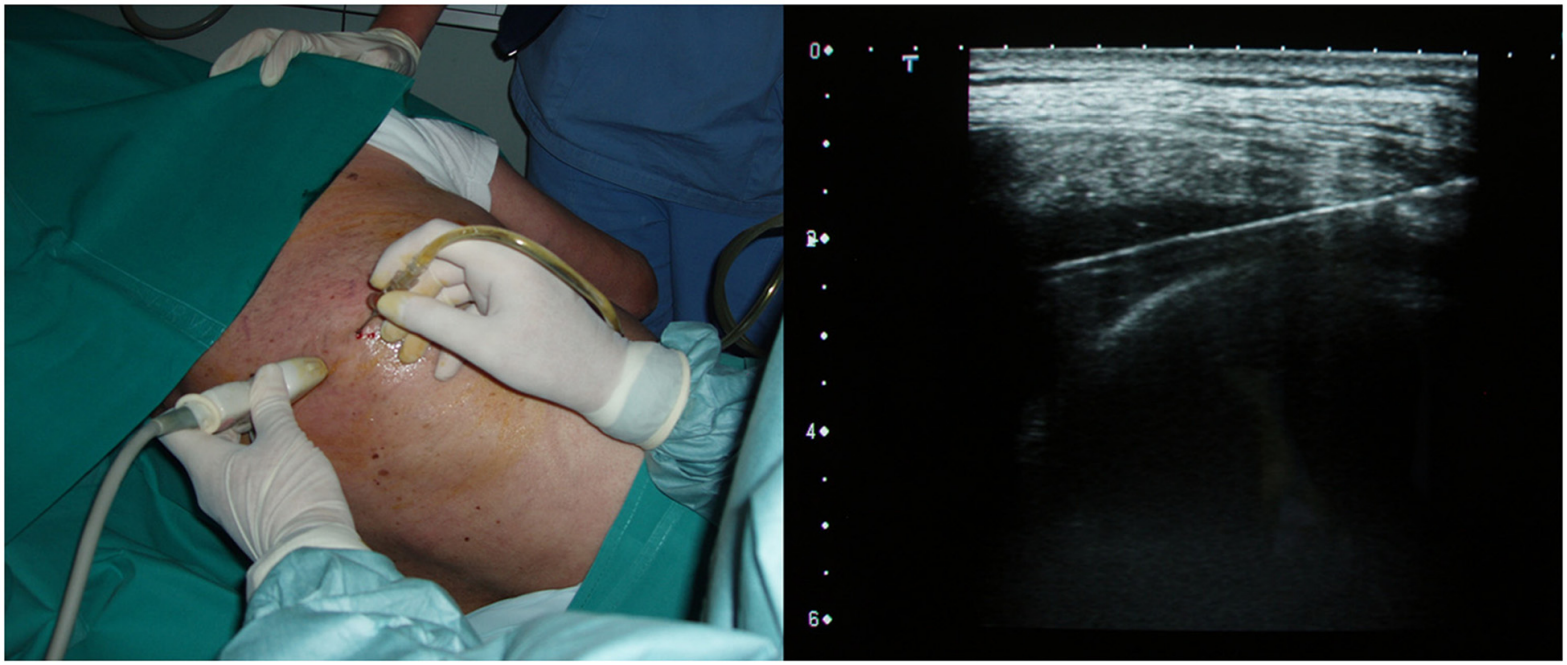
**Thoracentesis: the operator places the linear probe transversally. **A small pleural effusion in the costophrenic sinus appears as a transonic triangle bounded laterally by the diaphragm and the chest wall. Puncture site is identified as the apex of this triangle. An in-plane approach allows the Veres needle to be followed throughout the entire procedure.

Intercostal scans were obtained over the postero-lateral costophrenic sinus immediately above the diaphragm with the probe held longitudinally between the mid and posterior axillary lines. The sinus was reproduced in the echographic image as a transonic triangle bounded by the diaphragm and chest wall. Puncture site was identified at the apex of this triangle and an in plane approach was used. The needle was kept under constant vision and followed while entering the pleural space with a safe axis of penetration and a path almost parallel to the thoracic wall (Figure 3).

**Figure 4:**
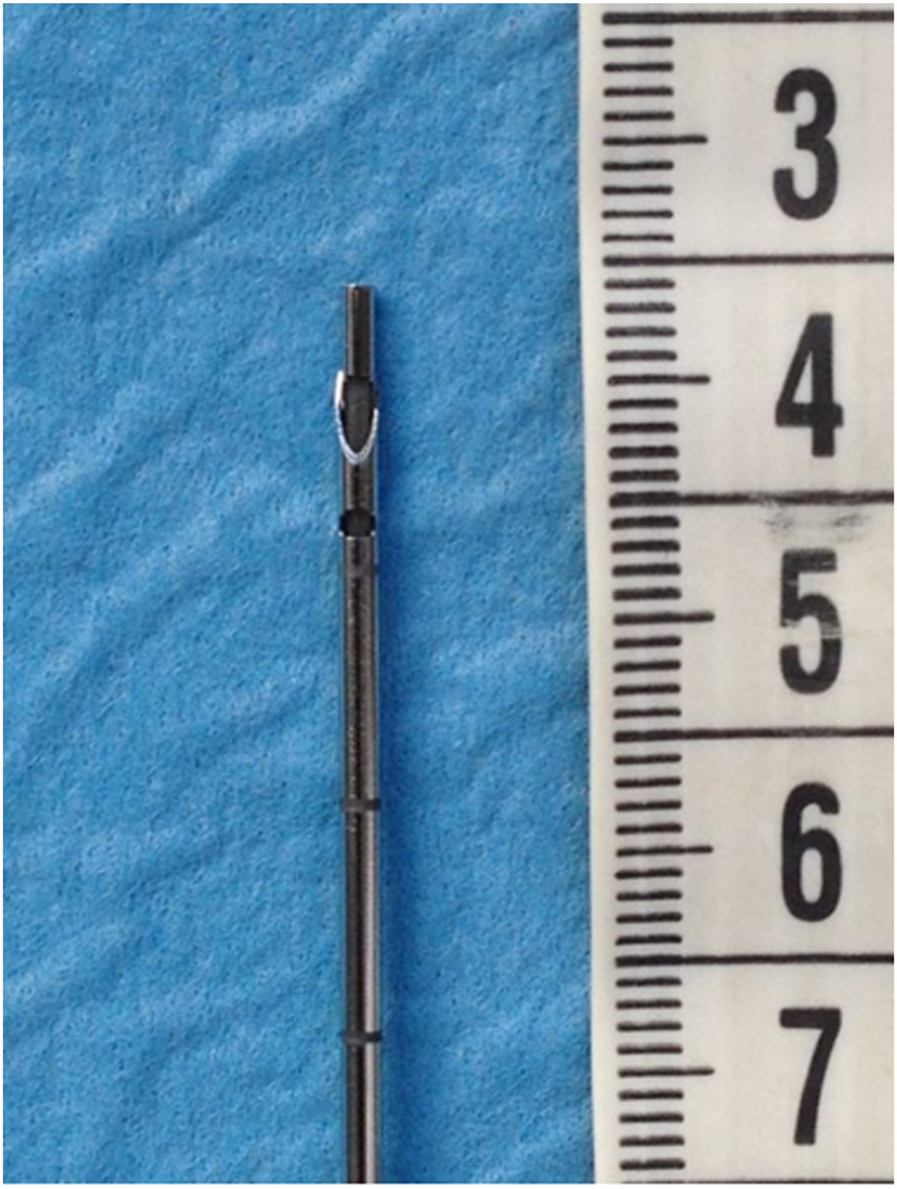
15-gauge Veres needle: detail of the tip.

For thoracentesis, a 15-gauge Veres needle with outside diameter of 1.8 mm (Toraset, Bioservice S.p.a., Poggio Rusco MN, Italy) was used. This triple-sharpened needle allows easy penetration while crossing thoracic wall whereas once in the pleural space a smooth cannula is inserted preventing traumatic parenchymal lesions (Figure 4). The Veres needle assembly included: a three-way stopcock, a large syringe (60 ml) and a bag (2000 ml). This system allows fluid drainage without syringe disconnection.

**Figure 5:**
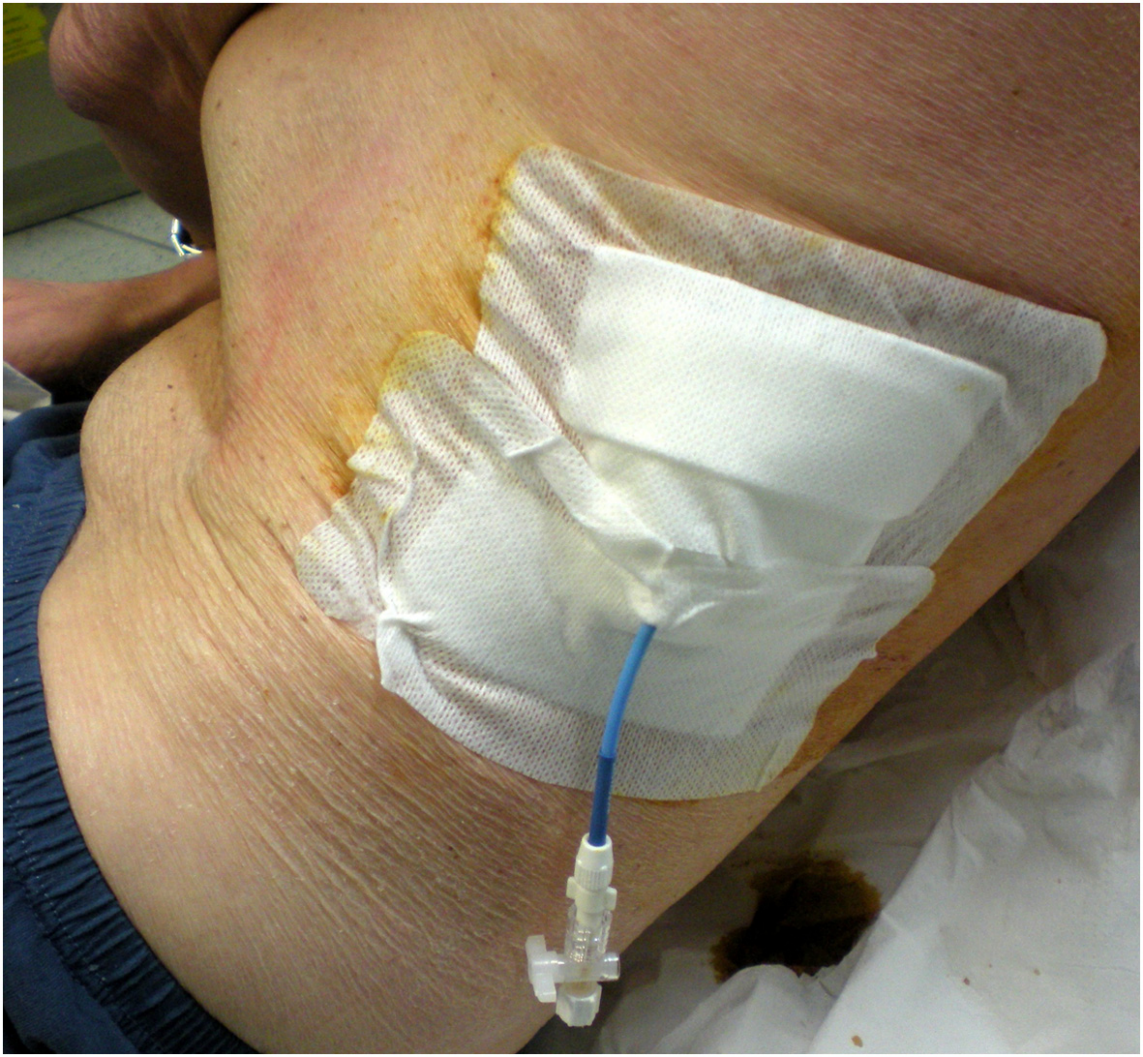
**Catheter drainage: the pigtail catheter is inserted in a very low, lateral position (between mid and posterior axillary lines). **In this way, it is not compressed by the patient lying in dorsal decubitus and may easily drain the pleural effusion.

Pigtail catheters were inserted using the Seldinger technique. Local anesthesia and needle puncture (18G needle) were carried out under constant echographic monitoring, as described above, through the lateral costo-phrenic sinus (Figure 5).

A 0.038 inch J-point guidewire was introduced through the needle in the pleural space, then two sequential dilators (8 and 12 Fr) and finally the Pigtail (Jinro 10.3 F, Boston Scientific).

Fluid drainage was suspended when one or more of the following conditions occurred: 1) over 2 L of fluid removed, 2) onset of chest pain, 3) onset of cough, 4) vasovagal event, 5) onset of dyspnoea, 6) haemorrhage. Each of these events was considered as a procedural complication if they necessitated procedure suspension. Instead we considered separately, and not as complications, local pain and cough if only transitory and readily disappearing when slowing down fluid aspiration.

Pain was defined as more than minor discomfort after sonographically administered anesthesia. Cough was considered excessive when it was so important to interfere with the running of a linear procedural. A vasovagal event was defined as sensation of light headness, diaphoresis, hypotension, or sudden transient loss of consciousness followed by recovery. Dyspnoea was characterized by unpleasant sensation of breathlessness with or without tachypnoea or oxygen desaturation.

We arbitrarily considered as post-procedural complications adverse events occurred within 24 hours of procedure completion. They included: pneumothorax (PNX), haemothorax, re-expansion pulmonary oedema, vasovagal event and late onset chest pain.

PNX was considered a complication of thoracentesis and was defined as new air demonstrated in the anterior pleural space on a post-procedural supine chest sonography [10]. In case of catheter drainage, PNX was considered a complication only if echographically present after complete fluid aspiration, if progressive and symptomatic, and if provoked by inspective needle contact with lung parenchyma, since the Seldinger technique often allows a moderate quantity of trans-parietal air to enter the pleural space. Sonographic signs of PNX were described in a previous work [11] and may be found in literature [12,13]. Post-procedural chest radiographs were not used according to literature [14,15] and since in ours and other groups experience chest sonography is more sensitive than chest X ray in detecting small pneumothoraces [11].

Thoracic Computed Tomography was performed according to clinical indications (e.g. diagnostic work-up, ultrasonographic evidence of PNX or haemothorax).

Acquisition time of the pleural space and procedural carry out time were analyzed. This time was calculated from the anesthetic puncture to the first appearance of fluid in the syringe. All unsuccessful drainages were registered.

A statistical analysis was performed using SPSS v.13.0 for Windows software (Chicago IL, USA). Results were expressed as mean ± standard deviations and categorical data. The differences between groups were calculated using independent *t*-test, Mann-Withney and Wilcoxon non parametric test, where appropriate. A *p* less than 0.05 was considered significant.

## Results

A total of 106 patients were enrolled. One hundred and one separate thoracenteses were performed on 76 patients (some patients underwent sequential thoracenteses) and 30 pigtail catheters were inserted in 30 patients (for a total of 131 pleural procedures in 106 patients enrolled). The two groups undergoing thoracentesis and pleural drainage were comparable for age, coexisting disease, and presenting symptoms with the exception of dyspnoea more present in the pleural drainage group. Demographic and epidemiological data of the patients are listed in Table 1.

**Table 1:** Demographic and clinical characteristics of the enrolled patients

**Patients’ characteristics (first admission)**	**n.**	**%**
**Thoracentesis**		
Total number of patients	76	
Males	47	61.8
Non invasive ventilation	3	3.9
Invasive ventilation	1	1.3
**Symptoms at admission**		
Chest pain	19	25
Fever	2	2.6
Dyspnea	54	71
Cough	9	11.8
**Chest drainage**		
Total number of patients	30	
Males	21	70
Non invasive ventilation	3	10
Invasive ventilation	0	
**Symptoms at admission**		
Chest pain	18	60
Fever	10	3.3
Dyspnea	28	93.3
Cough	5	16.6

Sixty eight patients (64%) were males with mean age of 67.2 ± 13 years and thirty eight (36%) were females with mean age of 70.5 ± 15 years (p: n.s.).

Seventy seven procedures were performed on males (58.7% of 131 procedures) and fifty four on females (41.3% of 131 procedures).

Procedure feasibility was 100% and in every case it was possible to follow the needle point entering the pleural space obtaining fluid.

Ninety four effusions were classified as medium (71.8%), 10 were small (7.6%) and 27 were large or massive fluid collections (20.6%). After drainage, 90.8% (119) of the effusions were minimal and 12 (9.2%) were small. Definitive diagnosis and cause of all pleural effusions are shown in Table 2, where fluid is classified as transudation or exudation [9], and according to the nature of disease. Twenty six effusions were transudates (19.8%) and 105 were exudates (80.2%). Seventy eight (59.5%) were transonic effusions and 53 were complex effusions (37 corpuscolated, 8 fibrinous and 8 areolar, 28.2%, 6.1% and 6.1%, respectively). A complex sonographic pattern was statistically associated with the exudative nature of the effusion. Heart failure effusions were seen as transonic effusions in 95% of cases.

**Table 2:** Definitive diagnosis and cause of pleural effusion

	**N**	**%**
**Total**	131	
**Transudate**	26	19.8
Congestive heart failure	26	19.8
**Exudate**	105	80.2
Neoplastic	28	21.4
Parapneumonic	54	41.2
Trauma	16	12.2
Connective tissue disease	7	5.3

Ninety eight thoracenteses (97%) and all catheter drainages were successfully completed. Twenty four pleural interventional procedures (18.3%) were stopped after reaching the maximal volume of 2000 ml of drained fluid. Four thoracenteses were stopped because of the appearance of complications.

In particular, two patients developed cough (1.5%), one patient PNX (0.7%) and one a vasovagal event (0.7%). No pigtail drainage was stopped for onset of complications.

Table 3 shows procedural and post-procedural complications.

**Table 3:** Characteristics and events (procedural and post-procedural complications) of pleural punctures

	**N**	**%**
Pleural punctures (thoracentesis + catheter drainage)	131	
Successfully completed pleural punctures	127	97
Punctures stopped for maximal fluid drainage (2000 ml)	24	18.3
**Procedural complications**	4	3
Chest pain	0	
Cough	2	1.5
Vasovagal event	1	0.7
Dyspnea	0	
Hemorrhage	0	
Pneumothorax	1	0.7
**Postprocedural complications**	2	1.5
Pneumothorax	1	0.7
Haemothorax	0	
Re-expansion pulmonary edema	0	
Vasovagal event	0	
Late onset chest pain	1	0.7

No puncture of thoracic nor abdominal organs, puncture of intercostal vessels nor intracavity/external bleeding was reported.

During 24 hours patient monitoring, we encountered only one chest pain (0.78% of completed procedures) which was partially explained by the intrinsic pleural disease (malignancy), and a case of PNX (0.78%) in a patient with bullous emphysema admitted for staphylococcal pneumonia. We reported no cases of haemothorax, no re-expansion pulmonary oedema and no late onset vasovagal event.

Minor symptoms as cough or pain not requiring suspension of the procedure were more frequent and not significantly correlated with either of the two procedures involved. Seventy three patients (55.7%) experienced cough and seventeen subjects (13%) reported at least one episode of transient pain.

Mean procedure time was 76 ± 9 seconds for thoracenthesis and 185 ± 46 seconds for drainage insertion (p < 0.05).

## Discussion

This study describes a different technique for echoguided thoracentesis including a case series with an analysis of success and complications. Major advantages of this technique are patient comfort, the possibility to be carried out in all types of patients, short procedural time and a reduction of risks and complications due to constant atraumatic needle puncture.

Thoracentesis is to date generally performed with the patient sitting at the edge of the bed and leaning forward with arms resting on a bedside table [4]. Lateral recumbent or supine positions are limited to patients unable to sit. The advantage of the above described technique is being easily performed in the lying position thus increasing patient comfort and reducing vasovagal syncope rate and being as well easily performed in patients with obligate decubitus for example in Intensive Care Unit beds.

The lateral recumbent or supine position with head and chest elevated at 30-45° allows fluid to accumulate in the deepest part of the pleural space following gravity as it would be in the “usual” position (patient sitting at the edge of the bed and leaning forward) making it easily detectable by ultrasound and available for pleural procedures even if in small quantity.

Lung sonography allows a preliminary estimate of effusion volume and characteristics and this is, in our opinion, important for clinical purposes [8]. A small effusion occupying the costo-phrenic sinus is not visible on chest radiography and is no more than 150 ml. A retroparietal effusion able to be seen in a single scan (a lateral height of 5–7 cm) corresponds to 500–600 ml of fluid. Any further scan necessary to include the entire effusion to be seen, adds about 500–600 ml. Therefore an effusion classified as massive (three or more scans, 21–24 cm in height) will refer to over 1800 ml of fluid. These data are in line with Reuss e coll. [16].

The echographic aspect of the effusion is important in the choice of draining system, its caliber, and in the timing of the procedure. In fact an exudative effusion and in particular an areolar complex effusion is most often associated to infective or neoplastic disease and usually requires a large bore drainage [8,17].

Most studies on echo-supported thoracentesis do not provide details on the technique used and are not able to associate patient comfort in the lying position with a complete real time visualization of each operating phase. Correct echographic guiding should allow constant monitoring of needle point position and this, for physical reasons, is only possible if the needle is positioned in the same scanning plane as the probe [18]. In the thorax in particular, this is only feasible if the probe is placed along an intercostal space with the needle penetrating inside the scan plane (in-plane). With the in-plane approach, the needle enters the skin at the side of the probe. Then the needle crosses the plane and the whole shaft is visualized as it progresses towards the target. For this reason, the costophrenic angle is the perfect position for this echoguided acquisition.

Iatrogenic PNX is the most common and important complication following pleural puncture [19]. Several risk factors have been identified as the type of needle used [20], the presence of mechanical ventilation, characteristics of the patient as the presence of pulmonary emphysema, operator experience and even the absence of an echographic guide [21,22]. It has been recently confirmed how programs of ‘best practice’ may reduce the risk of post-procedural PNX [23] and regarding this discussion we believe that in addition to operator training, it is important to standardize the materials and methods used. Following these recommendations, in fact, iatrogenic PNX rate, which is reported in literature to be between 5-10% and 28% [24-26], may be reduced to around 2% [23]. This risk reduction is particularly evident when using echography.

Other reported complications are not very significant both clinically and in terms of numbers (vasovagal events 3%, pain 5%, cough 24%), or are very rare (hemothorax, abdominal organ lesions) [23].

PNX can develop during thoracentesis if a communication between atmosphere and pleural space is established because of negative pleural pressure, that occurs when the needle for thoracentesis lacerates the lung and allows air to enter the pleural space from the alveoli, or if a rapid decompression of pleural space due to fluid removal lacerates the visceral pleura [14,19,22,26]. The latter occurrence is relatively common in patients with neoplastic and chronic effusions, and a chronically atelectatic lung.

In our series, we observed a case of PNX in a patient with lung cancer and a chronic large pleural effusion in whom at no time was the needle in contact with lung parenchyma. Another case of PNX developed as post procedural event in a patient with bullous emphysema and staphylococcal empyema. PNX presented thus as a complication in only 1.4% of cases. In both cases a direct needle contact with lung parenchyma was not seen and it is thus likely that their aetiology is not linked to a predictable procedural lesion but rather to an ‘ex vacuo’ mechanism. Their well documented aetiology is drainage- related rather than due to penetrating lung trauma or external air introduction [22]. In our cases echographic diagnosis was immediate and no drainage was necessary. In fact it has been reported that in *ex vacuo* PNX following thoracentesis, chest tube placement is not necessary in asymptomatic patients and is unlikely to provide clinical benefit [27].

In the group of patients treated with a pigtail catheter a small passage of air in the pleural space when inserting the guidewire took place if the effusion volume was less than 1500 ml and the intrapleural pressure presumably low. This often created a small anterior pneumothorax easily detected by lung ultrasonography and always drained through the inserted catheter without any clinical consequence. In no case there was evidence of a contact between the needle and lung parenchyma, echographic evidence of air filtration from the lung, or persistent or progressive PNX after air aspiration**.** For this reason it is not usually considered as complication of pleural puncture, and we considered it as a complication only in the two cases described.

It is possible that our technique reduced complications both because of the features of the Veres needle and for the constant direct real time view of the needle in the costophrenic sinus. Veres atraumatic needle was introduced in clinical practice in 1932 by Janos Veres [28] to create an artificial PNX. Curiously, its recent and actual use is almost entirely dedicated to laparoscopic procedures. To the best of our knowledge the only study considering this type of needle for thoracentesis is that by Jenkins et al. [29]. Our study is therefore the first study using Veres needle for echoguided thoracentesis procedures.

As far as cough onset is concerned, previous studies report a rate of 9 to 24% [21,30]. In our patients, transitory cough not requiring procedural stop was frequent (55.7%), and without difference among patients undergoing thoracentesis or pigtail catheter drainage, while the onset of cough as a complication leading to procedure interruption was only 1.5%. We agree with Jones et al. that the onset of persistent cough should represent an indication to stop the procedure [7]. In fact, cough onset is a risk factor for needle contact with lung parenchyma causing a possible lung lesion. Since in our proposed technique the needle point may easily be moved toward thoracic wall, any sudden patient movement may be anticipated becoming less dangerous.

Regarding vasovagal events, the literature indicates a rate of occurrence from 2 to 3.9% [31]. We report a much smaller rate of 0.7% possibly due to a more comfortable patient positioning. Presyncopal symptoms in one of our patients led to the interruption of the procedure.

No case of re-expansion pulmonary oedema (a complication of thoracentesis described in literature [32,33]) occurred in our series. Slow removal of pleural fluid associated with the previously defined limits of the procedure allowed us to reduce the risk of this complication.

The small number of complications reported in our series is, in our opinion, due to the particular choice of the point of puncture, the real time echographic acquisition and following of the needle point through all steps, and again the use of a Veres needle.

Recently, Patel and coll. highlighted the importance of US guidance for thoracentesis in terms of lower total hospital stay costs and lower incidence of PNX and hemorrhage [34]. Although Veres needle assembly is more expensive than commonly used needles, the low rate of complications observed in this study makes our procedure cost-effective, and it is possible that the low rate of complications observed in the present study can match the cost of the needle in comparison with other reports.

This is an observational study describing a new technique to perform thoracentesis. Although in literature there are no prospective studies comparing ‘blind’ to ‘echoguided’ pleural drainage, evidence exists regarding how echographic support reduces thoracentesis risks and complications both in the spontaneously breathing patient and in the mechanically ventilated patient [23,34-36]. The purpose of this study, in fact, was not a comparison between blind and echoguided techniques as advantages of the second one have already been well appointed and would not be ethically supportable at this time.

## Conclusions

This work highlights the safety and efficacy of a real time echo-monitored thoracentesis, in terms of more comfortable patient position, easier approach for the operator, very low rate of complications and short acquisition time of the pleural space.

Our major complications rate (PNX, dry tap, hemothorax, visceral lesions) are less or almost comparable to the best results of recent studies employing ultrasound assisted or guided needling of the pleural space.

The most useful advantage of the proposed technique of costophrenic cannulation is the access of the needle in plane with ultrasonic beam and parallel to the chest wall. In this way, the entire procedure is imaging controlled and the needle tip is under constant vision far from the lung (echo-monitored procedure).

Although the role of the Veres needle was not specifically addressed in this study, its documented safety in the abdominal field may logically be applied to the thorax and this, in our opinion, justifies its use.

## Abbreviations

PNX: Pneumothorax.

## Competing interests

The authors have reported to *Multidisciplinary Respiratory Medicine* that no potential conflicts of interest exists with any companies/organizations whose products or services may be discussed in this article.

## Authors’ contributions

GS, AS and RI equally contributed to the design of the study, performed procedures, contributed to data collection and writing the manuscript. SS contributed to the writing of the manuscript and language revision. GMC contributed to the analysis and interpretation of data. SV contributed to the review of the manuscript and to final approval of the version to be published. All authors read and approved the final manuscript.
